# Heparanase Increases Podocyte Survival and Autophagic Flux after Adriamycin-Induced Injury

**DOI:** 10.3390/ijms232012691

**Published:** 2022-10-21

**Authors:** Hanan Abu-Tayeh Suleiman, Shereen Said, Haya Ali Saleh, Aviva Gamliel-Lazarovich, Eyas Haddad, Irina Minkov, Yaniv Zohar, Neta Ilan, Israel Vlodavsky, Zaid Abassi, Suheir Assady

**Affiliations:** 1 Department of Nephrology and Hypertension, Rambam Health Care Campus, Haifa 3109601, Israel; 2 Rappaport Faculty of Medicine, Technion—Israel Institute of Technology, Haifa 3109601, Israel; 3 Department of Pathology, Rambam Health Care Campus, Haifa 3109601, Israel; 4 Cancer and Vascular Biology Research Center, Rappaport Faculty of Medicine, Technion, Haifa 3109601, Israel; 5 Department of Laboratory Medicine, Rambam Health Care Campus, Haifa 3109601, Israel

**Keywords:** heparanase, Adriamycin nephropathy, autophagy, podocytes, cell viability, glomerular filtration barrier, Roneparstat

## Abstract

The kidney glomerular filtration barrier (GFB) is enriched with heparan sulfate (HS) proteoglycans, which contribute to its permselectivity. The endoglycosidase heparanase cleaves HS and hence appears to be involved in the pathogenesis of kidney injury and glomerulonephritis. We have recently reported, nonetheless, that heparanase overexpression preserved glomerular structure and kidney function in an experimental model of Adriamycin-induced nephropathy. To elucidate mechanisms underlying heparanase function in podocytes—key GFB cells, we utilized a human podocyte cell line and transgenic mice overexpressing heparanase. Notably, podocytes overexpressing heparanase (H) demonstrated significantly higher survival rates and viability after exposure to Adriamycin or hydrogen peroxide, compared with mock-infected (V) podocytes. Immunofluorescence staining of kidney cryo-sections and cultured H and V podocytes as well as immunoblotting of proteins extracted from cultured cells, revealed that exposure to toxic injury resulted in a significant increase in autophagic flux in H podocytes, which was reversed by the heparanase inhibitor, Roneparstat (SST0001). Heparanase overexpression was also associated with substantial transcriptional upregulation of autophagy genes *BCN1, ATG5,* and *ATG12*, following Adriamycin treatment. Moreover, cleaved caspase-3 was attenuated in H podocytes exposed to Adriamycin, indicating lower apoptotic cell death in H vs. V podocytes. Collectively, these findings suggest that in podocytes, elevated levels of heparanase promote cytoprotection.

## 1. Introduction

Glomerular diseases are the leading cause of end-stage kidney disease [1]. Deep understanding of the pathogenesis of this heterogeneous group of chronic kidney diseases is critical for the development of new therapeutic approaches [2]. The integrity of the glomerular filtration barrier (GFB) is essential for maintaining normal kidney function. Remarkably, GFB is enriched with anionic heparan sulfate proteoglycans, which are an essential component of extracellular matrices and cell surfaces [3,4,5,6]. In mammals, heparanase is the only known endo-β-D-glucuronidase capable of cleaving heparan sulfate (HS) side chains [7,8]. Its activity affects both physiological and pathological processes [9,10]. However, the enzyme’s involvement in the pathogenesis of glomerular kidney diseases remains elusive [11,12,13]. Upregulation of heparanase expression and/or activity was reported in patients with various kidney diseases and after kidney transplantation [14,15,16]. It was also observed in experimental models of renal diseases in rodents [17,18,19,20,21,22,23,24], including Adriamycin-induced nephropathy (ADR-N), a well-characterized and established model for progressive proteinuric kidney disease [25]. Moreover, heparanase null mice and mice treated with heparanase inhibitor (PI-88) or neutralizing antibodies had mild kidney damage and proteinuria in experimental glomerular disease [18,26,27].

Recent study from our laboratory [28] demonstrated that Adriamycin toxicity resulted in massive albuminuria in BALB/c wild-type control mice, accompanied by severe glomerular and tubular injury, foot process effacement, and reduced expression of podocyte proteins implicated in GFB. Surprisingly, transgenic mice overexpressing human heparanase (*hpa*-TG) maintained glomerular function and structure after Adriamycin injection, suggesting a nephroprotective effect of heparanase in ADR-N [28]. Of note, the above-described conflicting results might be attributed to several factors, among others: (1) mouse models were of diverse genetic backgrounds, (2) heparanase expression was constitutively or temporally upregulated in some models, and (3) heparanase in available mouse models is expressed or knocked-out in all body tissues, rather than in a tissue- or cell-specific manner.

Thus, we hypothesized that in response to injury, constitutive overexpression of heparanase will have a direct protective effect on glomerular cells. Here, we focused on podocytes—terminally differentiated glomerular epithelial cells—because of their fundamental role in maintaining permselectivity and structural integrity of the GFB [29,30]. To this end, we employed both mouse models and a human podocyte cell line as experimental platforms [31]. We provide evidence that survival and viability of heparanase-overexpressing podocytes following Adriamycin-induced injury was markedly enhanced. Protective mechanism was associated with upregulation of autophagic flux both in vitro and in vivo, pari passu with attenuated apoptosis.

## 2. Results

### 2.1. Podocytes Overexpressing Heparanase Are Resilient to Cellular Injury In Vitro

To examine the effect of heparanase on podocytes irrespective of either systemic or local glomerular influences, an in vitro approach was employed. Undifferentiated AB8/13 cells (P) were infected with either an empty vector (Vo), or a vector containing human heparanase gene construct (H). Cells were allowed to differentiate for two weeks at 37°C. As shown in Supplementary Figure S1, differentiated Hepa (H) podocytes expressed and converted the latent 65 kDa heparanase into 50 kDa and 8 kDa subunits, which are known to comprise the active enzyme [8,9].

**Figure 1:**
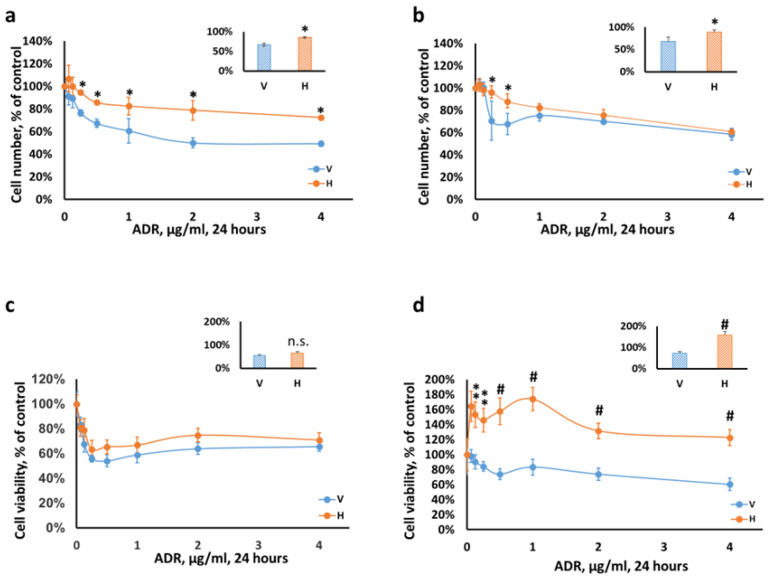
Heparanase protects podocytes from Adriamycin-induced cell death. Podocytes, V and H cells, were seeded in 96-well plates. (**a**,**c**) Undifferentiated or (**b**,**d**) differentiated podocytes, were exposed to different concentrations of Adriamycin (ADR) for 24 h. Cell number (**a**,**b**) was determined using methylene blue assay. Cell viability (**c**,**d**) was determined using Neutral Red Assay Kit. Results are expressed as percent of control for each cell type, i.e., the ratio of treated cell number to control untreated cells. The results are expressed as the means ± SEM of 3 independent experiments, quadruplicates in each experiment. Inserts: Cell survival/viability at ADR 0.5 µg/mL. H, heparanase overexpressing podocytes; V, mock-infected podocytes; n.s., not significant. * *p* ≤ 0.05, ** *p* ≤ 0.01, # *p* ≤ 0.001 H vs. V.

To induce injury, podocytes were incubated with Adriamycin for 24 h. We found that heparanase had a protective effect on both undifferentiated and differentiated podocytes (Figure 1). In the undifferentiated state, heparanase had a prominent pro-survival effect observed along all tested Adriamycin concentrations (0.25–4 µg/mL; Figure 1a). When cells exit the cell cycle and differentiate, they became more susceptible to toxic Adriamycin injury while retaining a significant advantage in cell number at a narrow Adriamycin concentration window of 0.25 and 0.5 µg/mL (26% and 20% higher in Hepa (H) vs. control (V) cells, respectively; Figure 1b). Likewise, exposure of cultured podocytes to hydrogen peroxide, an additional model of injury, resulted in a similar higher survival index of differentiated Hepa (H) compared with control (V) podocytes (15% at 0.5 mM; Supplementary Figure S2a). However, cell number is not directly related to cell viability. Therefore, we utilized the neutral red assay to measure podocyte viability (Figure 1c,d). The principle of this assay is based on the ability of living cells to incorporate the chromophore into their lysosomes. Remarkably, the uptake of neutral red into differentiated podocytes overexpressing heparanase (H) was significantly superior to control (V) cells at all tested Adriamycin concentrations, implying an increased lysosomal activity of viable cells. In contrast, uptake of neutral red by undifferentiated podocytes was comparable between Hepa (H) and control (V) cells. On the basis of these studies, we used an Adriamycin concentration of 0.5 µg/mL for subsequent experiments.

Together, these data indicate a possible protective role of heparanase in differentiated podocytes following exposure to Adriamycin, possibly by a mechanism related to lysosomal activity.

### 2.2. Heparanase Enhances Autophagic Flux following Podocyte Injury

Next, we investigated potential rescue mechanisms that might explain the survival and viability advantage conferred by heparanase. We addressed autophagy as a protective pathway because of its link to lysosomes and maintenance of terminally differentiated cells like podocytes [32,33]. We utilized (1) the conversion of microtubule-associated protein 1A/1B-light chain 3 (LC3)-I to LC3-II through phosphatidylethanolamine conjugation, and (2) the degradation of p62 (an autophagy substrate), to monitor autophagic activity [34].

**Figure 2:**
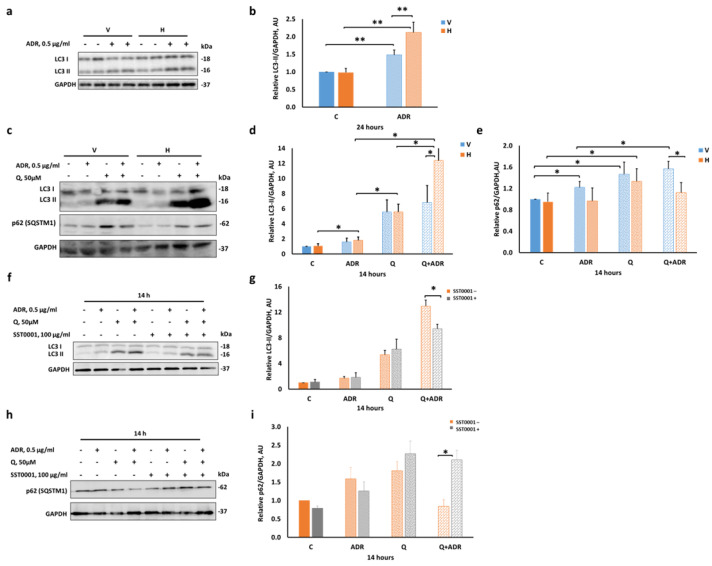
Heparanase enhances autophagy in differentiated podocytes after Adriamycin-induced injury. (**a**,**b**) Mock-infected (V) and heparanase overexpressing (H) differentiated podocytes were treated with 0.5 µg/mL Adriamycin (ADR) for 24 h. (**c**–**i**) Autophagic flux was assessed by incubation of cells with 50 µM chloroquine (Q) ± 0.5 µg/mL ADR for 14 h. (**f**–**i**) Hepa (H) cells treated with 50 µM chloroquine (Q) ± 0.5 µg/mL ADR ± 100 µg/mL SST0001 heparanase inhibitor, for 14 h. Whole-cell lysates were subjected to SDS-PAGE and immunoblotting for LC3 or p62 (also called SQSTM1) with GAPDH as a loading control. Quantification of LC3-II (16 KDa) (**b**,**d**,**g**) and p62 (**e**,**i**) normalized to GAPDH. (**a**,**c**,**f**,**h**) Representative immunoblots of LC3 and p62. Results are expressed as the means ± SEM, *n* = 3–6 independent experiments. * *p ≤* 0.05, ** *p ≤* 0.01.

Differentiated control (V) and Hepa (H) podocytes were treated with 0.5 µg/mL Adriamycin for 24 h and cell extracts were subjected to immunoblotting (Figure 2a). LC3-II levels were increased following Adriamycin treatment in both control (V) and Hepa (H) cells vs. untreated cells (1.43- and 2-fold, respectively; Figure 2b). Of note, heparanase overexpression resulted in higher levels of LC3-II in podocytes relative to control (V) cells under stress.

Because autophagy is a dynamic process, the increase in LC3-II level could reflect either increased formation or reduced degradation of autophagosomes. Chloroquine, an inhibitor of autolysosomal degradation, was applied to distinguish between these two possibilities. We observed that activation of autophagy by Adriamycin was time-dependent, resulting in a significant increase of LC3-II levels after 14 and 24 h after treatment with both Adriamycin and chloroquine (Supplementary Figure S3). However, we noticed that chloroquine was toxic to cells after an extended incubation period (24 h), especially when combined with Adriamycin. Therefore, further experiments were limited to a maximum duration of 14 h.

As expected, the addition of 50 µM chloroquine led to comparable accumulation of LC3-II (6-fold) and p62 (1.6-fold) proteins in both control (V) and Hepa (H) podocytes (Figure 2c–e), reflecting the basal autophagic flux in cultured podocytes. In the absence of chloroquine, Adriamycin treatment for 14 h increased LC3-II, with no change in the levels of p62, in Hepa (H) podocytes compared with untreated counterparts, suggesting autophagy activation (Figure 2c–e). In comparison, control (V) podocytes exhibited no change in LC3II levels following Adriamycin treatment but showed a noticeable increase in p62 levels vs. untreated V podocytes. Remarkably, 0.5 µg/mL Adriamycin or 0.5 mM H_2_O_2_ (an oxidative stress insult) combined with chloroquine showed higher LC3-II accumulation than in the chloroquine group and in H podocytes compared with V podocytes (Figure 2c–d and Supplementary Figure S2b, respectively). Furthermore, the chloroquine–Adriamycin combination caused significant increase in p62 levels in V podocytes compared with Hepa (H) podocytes (Figure 2c,e).

Collectively, these results indicate that constitutive heparanase overexpression significantly activates autophagic flux in podocytes in response to Adriamycin treatment. This was revealed by increased LC3II levels in the presence of the inhibitor chloroquine vs. LC3-II levels without the inhibitor and by the differential LC3II levels between control (V) and Hepa (H) podocytes.

To elucidate the specificity of heparanase effect, SST0001 (Roneparstat), a chemically modified heparin and inhibitor of heparanase, was added to the culture media of Hepa (H) cells four hours before and during various treatments. As shown in Figure 2f–i, SST0001 reversed the effect of heparanase on autophagic flux in podocytes, exemplified by reduction of LC3II levels in Adriamycin and chloroquine group and elevation of p62 levels, resembling the pattern observed in control (V) podocytes (Figure 2d,e; Adriamycin and chloroquine groups). Notably, the heparanase inhibitor did not affect the basal autophagic flux in these cells.

### 2.3. Heparanase Upregulates Autophagy Early after Induction of Injury

**Figure 3:**
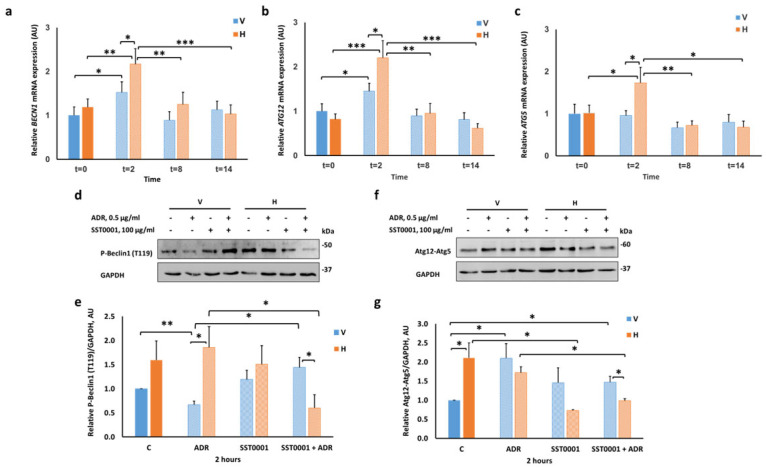
Heparanase overexpression increases podocyte autophagic salvage pathway early in response to Adriamycin. (**a**–**c**) RT-qPCR of *BECN1, ATG5*, and *ATG12* mRNA levels in podocytes (solid blue bars–V control, solid orange bars-H control) at 0, 2, 8, and 14 h after Adriamycin (ADR)-induced injury (light shaded bars). Values were normalized to *B2M.* Significant changes were observed at 2 h after injury. (**d**–**g**) V and H differentiated podocytes were treated with 0.5 µg/mL ADR ± 100 µg/mL SST0001 for 2 h. Whole cell lysates were subjected to SDS-PAGE and immunoblotting for phosphorylated Beclin1 at Thr119 (T119) or Atg12-Atg5. GAPDH was applied as a loading control. Quantification of P-Beclin1 (T119) (50 KDa) (**e**) and Atg12-Atg5 (60 KDa) (**g**) normalized to GAPDH. (**d**,**f**) Representative immunoblots of P-Beclin1 (T119) and Atg12-Atg5, respectively. Results are expressed as the means ± SEM, *n* = 3–6 independent experiments. * *p* ≤ 0.05, ** *p* ≤ 0.01, *** p ≤ 0.001.

To further explore the interplay between autophagy and heparanase, we first addressed the temporal changes in autophagy-related gene expression. To this end, podocytes were grown to subconfluency and then exposed to 0.5 μg/mL Adriamycin at various time points: 2, 8, and 14 h. RNA was extracted, and gene expression was quantified using RT-qPCR. Specifically, we tested the autophagy inducer BECN1 and the autophagosome elongation essential markers, ATG5 and ATG12 [34,35]. As shown in Figure 3, expression of these genes was upregulated two hours after treatment with Adriamycin in Hepa (H) cells, significantly higher than in control (V) cells (Figure 3a–c). ATG5 mRNA levels were not affected by Adriamycin in control (V) cells (Figure 3c). Hence, these results represent an early response of autophagy genes to injury in podocytes overexpressing heparanase.

Further experiments were also performed to assess the response of the respective proteins, two hours after Adriamycin treatment. Figure 3d,e demonstrates that heparanase maintained high basal levels of phosphorylated Beclin1 at Thr119 after Adriamycin administration, which were decreased by incubation with the heparanase inhibitor SST0001. Conversely, we observed a marked reduction in the levels of phosphorylated Beclin1 in control (V) cells after Adriamycin, which was reversed by SST0001.

The expression of Atg12-Atg5 conjugate was distinct in Hepa (H) podocytes. We observed that heparanase maintained high basal levels of Atg12–Atg5 conjugates, as well as following Adriamycin treatment, both of which were reduced by SST0001 (Figure 3f,g). However, in control (V) podocytes, Atg12-Atg5 levels significantly increased after Adriamycin but were not affected by adding the heparanase inhibitor.

Moreover, we demonstrated a robust increase in Atg12 level in Hepa (H) podocytes treated with Adriamycin for two hours. Incubation of cells with SST0001 significantly reduced Atg12 levels at the basal state and following Adriamycin-induced injury (Supplementary Figure S4a,b).

**Figure 4:**
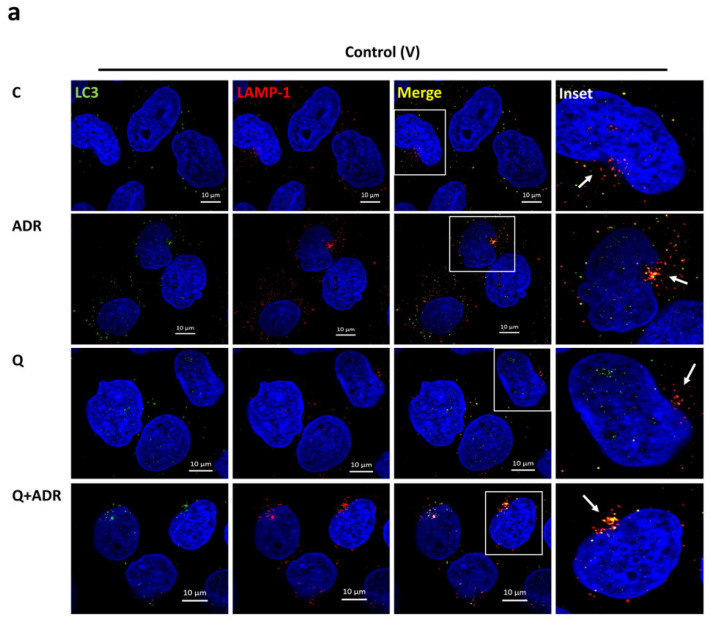

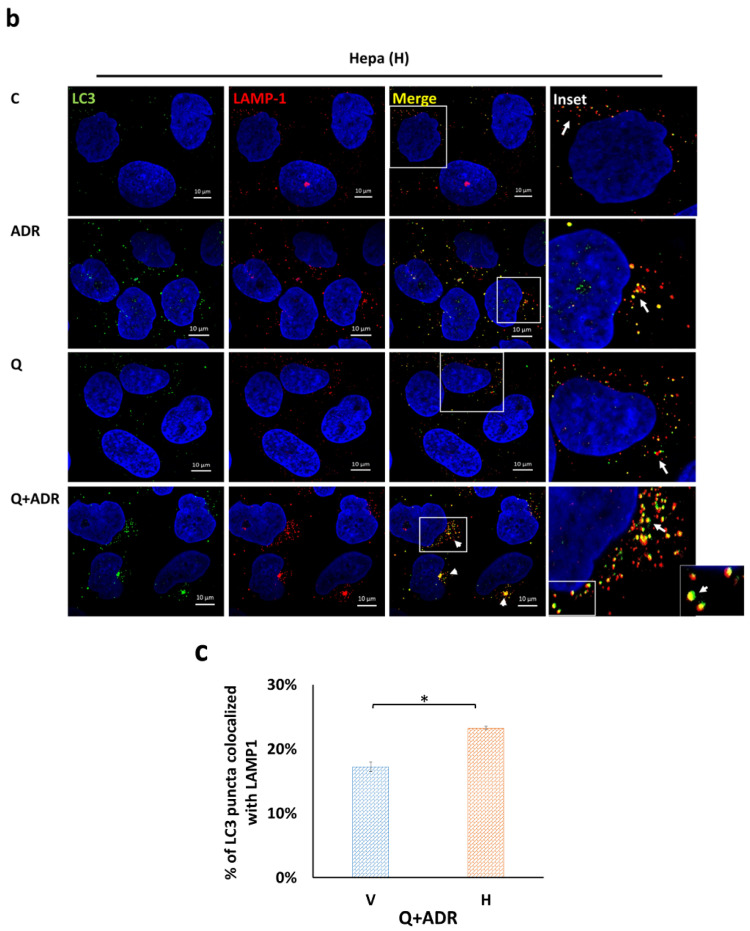
Immunofluorescence analysis reveals high autophagosome–lysosome colocalization in heparanase overexpressing podocytes. (**a**,**b**) V and H podocytes were double immunostained with anti-LC3 antibody (green) to identify autophagosomes, followed by staining with anti-LAMP1 antibody (red), a marker of the lysosomal membrane protein. Podocytes were treated with 0.5 µg/mL Adriamycin (ADR), with or without 50 µM chloroquine (Q), were fixed, permeabilized, and incubated with antibodies to LC3 and LAMP1 and counterstained with DAPI. Immunofluorescence staining shows the changes of autophagosomes–lysosome colocalization (yellow, white arrows) in various groups (×63 magnification). Boxed areas were zoomed; scale bar = 10 μm. (**c**) Quantitative analyses of LC3-LAMP1 colocalization staining (merge) are presented. Significant increase in autophagosome–lysosome colocalization was observed in H cells treated with Q + ADR compared with V cells. *n* = 2 per treatment group. At least 10 cells were scored for each condition. (Imaris software version 9.8.2, Oxford Instruments, Abingdon, UK). Results are expressed as the means ± SEM. * *p ≤* 0.05.

To further investigate the nature of the autophagic process in subcellular compartments, co-localization immunofluorescent staining was performed (Figure 4). For this purpose, Hepa (H) cells and control (V) cells were exposed to 0.5 µg/mL Adriamycin for 2 h, either with or without 50 µM chloroquine. As expected, the combination of Adriamycin and chloroquine led to LC3-II accumulation (Figure 4). In response to injury, co-staining for both LC3-II and lysosome-associated membrane protein 1 (LAMP1) was significantly higher in Hepa (H) compared with control (V) cells (23% vs. 17%, respectively).

To summarize, our results suggest that heparanase overexpression augments autophagy in podocytes, early in response to Adriamycin-induced injury, both at mRNA and protein levels.

### 2.4. Heparanase Increases Autophagic Flux in Mice following Adriamycin-Induced Nephropathy

**Figure 5:**
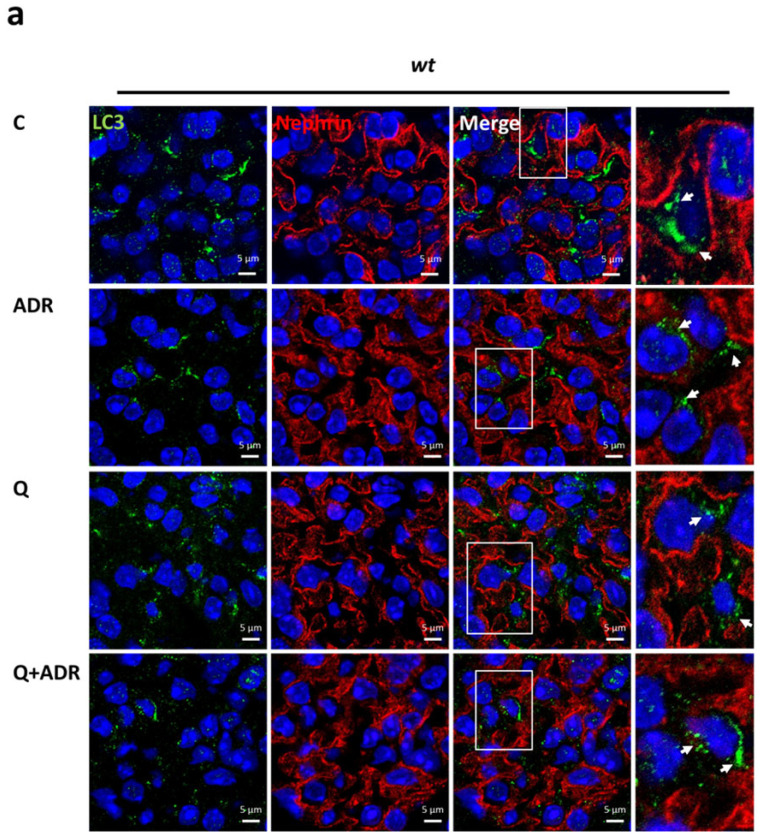

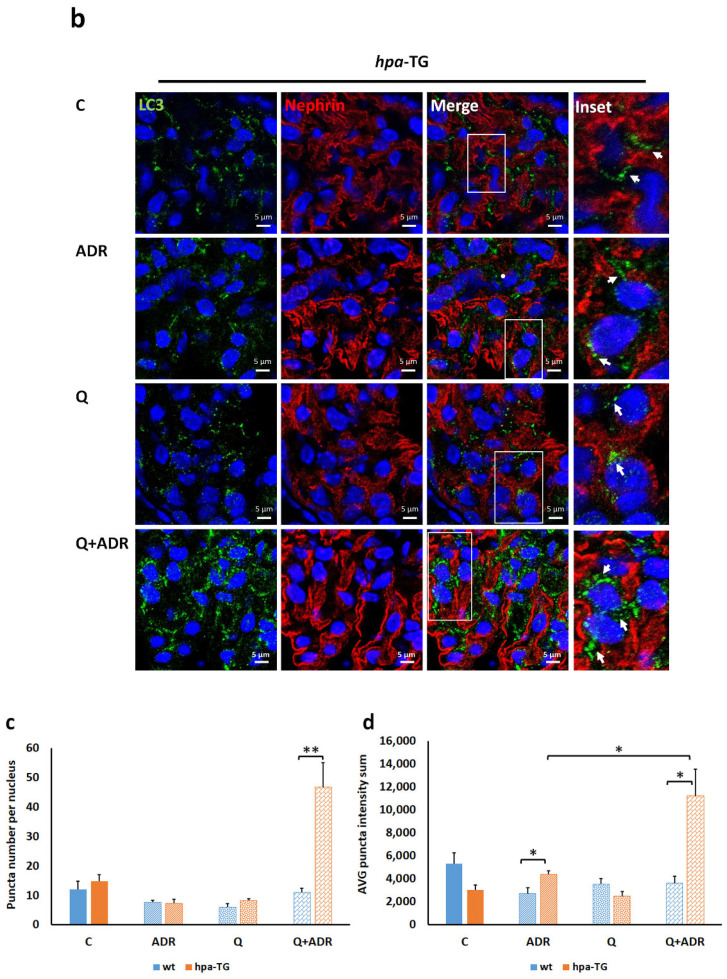
Increased autophagy in glomerular podocytes of heparanase transgenic mice (*hpa*-TG) compared with wild-type (*wt*) mice following Adriamycin-induced injury. Mice were treated with 10 mg/kg Adriamycin (ADR) or vehicle (C) for 3 days, without or with the addition of 40 mg/kg chloroquine (Q), 7 h before kidney harvest. 4 µm frozen sections of kidneys were immunolabeled for LC3 (green, white arrows) and nephrin (red) and counterstained with DAPI (blue). (**a**,**b**) Representative images. Scale bars represent 5 µm. (**c**) Quantitative analyses of LC3 puncta number and (**d**) fluorescence intensity in podocytes are presented. *n* = 3–5 mice per treatment group. For each treatment, 15–25 glomeruli were analyzed (Imaris software version 9.8.2, Oxford Instruments, Abingdon, UK). Results are expressed as the means ± SEM. * *p ≤* 0.05, ** *p ≤* 0.01.

To explore the role of autophagy in the preservation of podocytes in vivo, Adriamycin was injected into wild-type (*wt*) and *hpa*-TG mice as described previously [28], except that mice were sacrificed after 3 days (and not 14 days) following Adriamycin injection. To evaluate autophagic flux, chloroquine (40 mg/kg, i.p.) was administrated 7 h prior to kidney harvest. As demonstrated in Figure 5a, LC3-positive puncta were observed in nephrin-positive podocytes. When quantified, exposure to the combination of Adriamycin and chloroquine resulted in a significantly higher number of LC3-labeled puncta in *hpa*-TG podocytes compared with *wt* podocytes (4.2 folds; Figure 5b). Moreover, fluorescence intensity of LC3-labeled puncta was significantly higher in *hpa*-TG compared with *wt* mice treated with Adriamycin alone or in combination with chloroquine (1.6 and 3.1 folds, respectively; Figure 5c).

Taken together, these results strongly substantiate the role of heparanase in increasing autophagic flux in podocytes after injury in vivo, in accordance with the in vitro studies performed with the human podocytes.

### 2.5. Heparanase Attenuates Apoptosis in Podocytes

Analysis of the proteolytic processing of caspase-3 into active fragments, a hallmark of apoptosis, showed that Adriamycin markedly induced apoptosis in the differentiated parental (AB8/13) podocytes in a dose- and time-dependent manner (Supplementary Figure S5). Given the multifaceted effects of heparanase, we hypothesized that the enzyme may also attenuate apoptosis, contributing thereby to podocyte survival and viability after injury (Figure 1).

**Figure 6:**
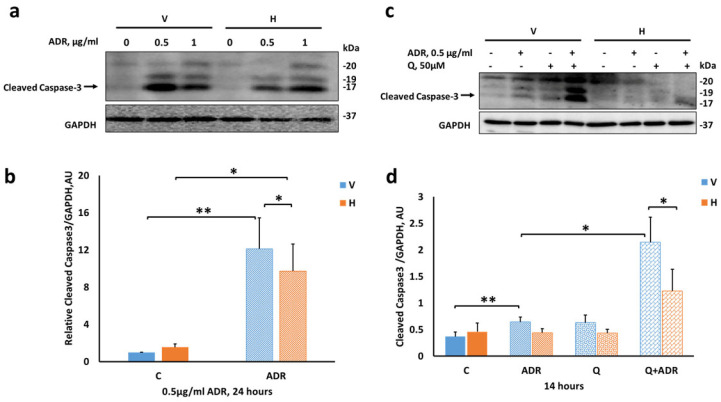
Adriamycin-induced apoptosis in differentiated podocytes. V and H differentiated podocytes were treated with either 0.5 or 1 µg/mL Adriamycin (ADR) for 24 h (**a**,**b**) or with 0.5 µg/mL ADR, 50 µM chloroquine (Q) or both for 14 h (c,d). Cell lysates were subjected to SDS-PAGE and immunoblotting of cleaved caspase-3 and GAPDH as a loading control. (**a**,**c**) Representative immunoblots of cleaved caspase-3. (**b**,**d**) Quantification of active cleaved caspase-3 (17 KDa) normalized to GAPDH at the indicated treatment concentrations. Results are expressed as the means ± SEM, *n* = 3 independent experiments. * *p ≤* 0.05, ** *p ≤* 0.01.

Therefore, we conducted the next experiments on differentiated control (V) and Hepa (H) cells. Cells were treated with 0.5 and 1 µg/mL Adriamycin for 24 h and cell extracts were subjected to immunoblotting. Adriamycin increased cleaved caspase-3 levels in both control (V) and Hepa (H) podocytes (Figure 6a,b). However, levels were significantly lower in Hepa (H) podocytes compared with control (V) cells.

Subsequently, combined incubation (14 h) of Adriamycin, in the presence or absence of the autophagy inhibitor chloroquine, uncovered marked differences between Hepa (H) and control (V) podocytes (Figure 6c,d). Adriamycin significantly increased cleaved caspase-3 in control (V) podocytes (1.78-fold vs. untreated cells, Figure 6c,d) but not in Hepa (H) podocytes, suggesting an attenuated apoptotic response by heparanase.

When autophagy was inhibited by chloroquine, control (V) podocytes exposed to Adriamycin exhibited profound apoptosis as reflected by significant increase in cleaved caspase-3 (Figure 6c,d). In comparison, under the same experimental conditions, cleaved caspase-3 levels determined in Hepa (H) podocytes were 43% lower than in control (V) podocytes (Figure 6b). These findings further support the notion that heparanase may protect podocytes via a mechanism that involves apoptosis.

## 3. Discussion

Genetically modified mice overexpressing the human heparanase gene provided a valuable platform for the initial assessment of heparanase role in kidneys both in health and disease [28,36,37,38]. Previously, we showed that *hpa*-TG mice were resistant to Adriamycin nephrotoxicity, resulting in minimal proteinuria and preserved podocyte morphology and expression of nephrin and podocin compared with wild-type control mice, despite the reduction in anionic charge of the GFB [28].

Here, we have confirmed and further extended these results, focusing on podocytes. Podocytes are polar epithelial cells that play an instrumental role in the GFB permselectivity [30]. Because the transgene in *hpa*-TG mice is driven by a constitutive β-actin promoter—i.e., expressed in all mouse tissues—the in vitro platform of human podocyte cell culture enabled us to investigate the function of heparanase in podocytes, which could be of relevance to human glomerular diseases.

The present study revealed, for the first time, potential molecular mechanism/s underlying heparanase protective role against podocyte injury induced by Adriamycin or hydrogen peroxide. Adriamycin^®^ (the brand name of the chemotherapeutic doxorubicin) is known to induce injury by direct toxic damage to all compartments of the GFB via multiple mechanisms involving mitochondrial dysfunction, oxidative stress, RAGE ligands, and DNA damage, among others [20,25,39,40,41,42,43,44]. Our findings clearly showed a significant decrease in the severity of Adriamycin cytotoxicity in heparanase-overexpressing podocytes versus their controls. Replicating podocytes, grown at 33 °C were resistant to insult throughout a wide range of drug concentrations. In contrast, cell number advantage was observed over a narrow range of Adriamycin concentrations in differentiated heparanase-overexpressing podocytes grown at 37 °C. Importantly, their viability was remarkably superior vs. differentiated mock-infected cells. This behavior is not surprising, because quiescent terminally differentiated cells are more susceptible to injury, yet heparanase overexpression granted viability advantage to surviving podocytes. Furthermore, we also found that heparanase promoted podocyte survival following damage induced by hydrogen peroxide, indicating that this phenomenon is general and applicable to various types of injury.

The mechanisms underlying the protective function of heparanase on podocytes are not entirely clear. Interestingly, heparanase augmented the lysosomal activity of viable cells, which was significantly higher in differentiated Hepa (H) podocytes compared with control (V) podocytes, at all tested Adriamycin concentrations. The lysosomes are end points of several degradation cellular pathways, including autophagy [45]. Autophagy is a critical housekeeping process that maintains cellular homeostasis. It consists of dynamic multi-step cellular pathways, responsible for the degradation of protein aggregates and damaged organelles, among others, and for the recycling of essential components [35,46,47,48]. Liu et al. [49] have previously shown that restoration of lysosomal activity could rescue advanced glycation end products (AGE)-treated podocytes and recover their autophagic activity.

**Figure 7:**
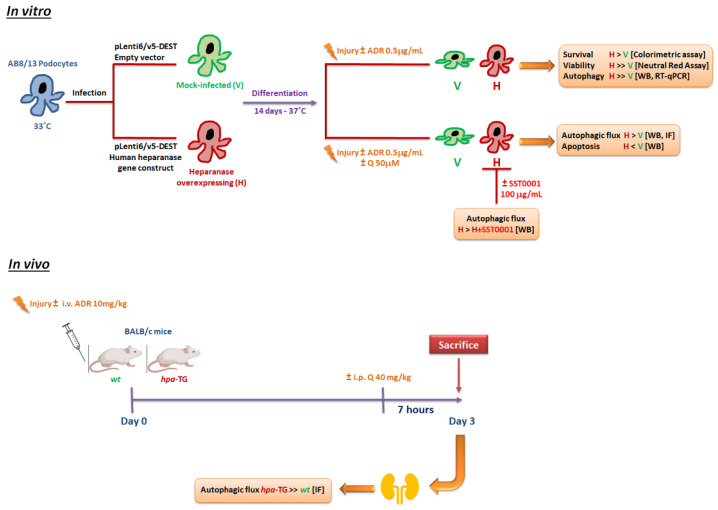
Potential mechanisms underlying the protective role of heparanase against Adriamycin-induced podocyte injury. A schematic illustration of the study design and results. ADR, Adriamycin; Q, chloroquine; *wt*, wild type; *hpa*-TG, transgenic mouse overexpressing human heparanase. Methods in square brackets: WB, Western blot; IF, immunofluorescence; RT-qPCR, reverse transcription-quantitative polymerase chain reaction.

In this study, we demonstrated that the protective effect of heparanase was associated with significant increase in autophagic flux and reduced apoptosis in vitro (Figure 7). Likewise, in experimental model of ADR-N, podocytes in kidneys of *hpa*-TG mice exhibited a significantly higher number of LC3-stained puncta compared with wild-type mice at early stages after injury and, importantly, prior to the expected onset of albuminuria, which was previously shown to occur 5–7 days after Adriamycin injection [25,28].

Induction of autophagy is thought to play an adaptive and protective mechanism against glomerular disease [46]. Differentiated podocytes have a high basal level of autophagy, which maintains their structural and functional integrity [32,50]. Our findings demonstrated that treatment with Adriamycin for 24 h significantly increased the levels of LC3-II in both control (V) and Hepa (H) podocytes, in line with reported findings in mouse podocytes treated with Adriamycin [51]. Importantly, we further showed that the increase in LC3-II was significantly higher in Hepa (H) than in control (V) podocytes. Increased cellular LC3-II levels may be indicative of autophagy stimulation. However, it should be complemented by displaying an enhanced autophagic flux, which represents the dynamic process of autophagy. Indeed, the lysosomal degradation inhibitor chloroquine augmented the accumulation of LC3-II mostly in Hepa (H) podocytes treated with Adriamycin for 14 h. However, no changes were observed in p62 relative to its basal levels, suggesting perhaps an increased rate of non-selective autophagic flux upon heparanase overexpression. In addition, and based on our findings with control (V) podocytes, we propose that autophagy may be impaired in V podocytes when treated with Adriamycin. Of note, p62 is a polyubiquitin-binding protein involved in many signal transduction pathways [52]. Hence, interpretation of its intracellular levels is complicated because it depends on transcriptional regulation as well as post-translational autophagic or other degradation events [47]. To further clarify the role of p62, additional experiments should be performed in the presence of proteasome or pan-caspase inhibitors.

The immunofluorescence analyses confirmed the increment in autophagic flux in Hepa (H) podocytes treated with Adriamycin. We demonstrated significant rise in the percentage of fusion dots, double immunostained for the autophagic marker, LC3, and the lysosomal marker, LAMP1, in Hepa (H) podocytes compared with control (V) podocytes, in the Adriamycin–chloroquine group. These findings were in line with immunostaining results in *hpa*-TG mice, suggesting autophagy as a common essential pathway in both human and murine cells, as well as in in vitro and in vivo experimental systems.

Treatment of Hepa (H) podocytes with SST0001, an enzymatic inhibitor of heparanase, reversed the effect of constitutive heparanase overexpression on autophagic flux, alluding to an enzyme-specific outcome. Interestingly, chloroquine treatment raises the lysosomal pH and thus can block heparanase enzymatic activity, which is pH sensitive [53]. This implies that heparanase may induce autophagy partially by other non-enzymatic activities. Previous studies reported that heparanase can also fulfill enzymatic-independent biological functions, including, among others, signal transduction [54,55] and gene transcription [56,57]. The 65-kDa latent heparanase could activate the serine/threonine kinase AKT, mediating endothelial cell migration and invasion in vitro [58]. Both active and inactive heparanase may enhance epidermal growth factor receptor phosphorylation, thereby increasing cell migration, cell proliferation, and colony formation [59]. Furthermore, a recent study revealed that heparanase enhances gastric cancer progression independently of its enzymatic activity by triggering the transcription factor EB (TFEB)-driven autophagy, in addition to the associated cell proliferation [60].

Our study extended this knowledge to podocytes, non-cancerous epithelial cells, which were resilient to induced injury in the presence of heparanase. To meet this goal, we speculated that heparanase activates autophagy at the early phase of injury. Indeed, significant increments in transcription levels of *BECN1, ATG5*, and *ATG12* were demonstrated two hours after induction of cell injury in Hepa (H) podocytes compared with control (V) podocytes. Moreover, constitutive heparanase overexpression maintained high basal levels of phosphorylated Beclin1 at Thr119 and Atg12-Atg5 conjugate proteins after Adriamycin administration. Previously, Gurkar et al. [61] showed that the Ser/Thr Rho kinase 1 (ROCK1) was activated upon nutrient deprivation and promoted autophagy by binding and phosphorylating Beclin1 at Thr119. Subsequently, specific dissociation of the Beclin1-Bcl-2 complex occurred without affecting the Beclin1-UVRAG interaction, suggesting that ROCK1 acts as a prominent upstream regulator of Beclin1-mediated autophagy and maintains a homeostatic balance between apoptosis and autophagy [61]. It has also been reported that death-associated protein kinase-1 (DAPK1) could phosphorylate the BH3-domain residue Thr119 of BECN1 and consequently abrogate BECN1-BCL2/BCL-XL interaction, facilitating autophagy initiation upon serum deprivation [62]. In neurons, other type of terminally differentiated cells, GPCR kinase 2-interacting protein-1 (GIT1) regulated the phosphorylation of Beclin-1 at Thr119, which eventually rescued cells from ischemia–reperfusion injury by promotion of mitophagy and inhibition of apoptosis [63]. Thus, we suggest that heparanase may involve one of the previous regulators, which initiate autophagy and reduce apoptosis after podocyte injury, warranting further investigation.

The association between increased autophagy and epithelial cell survival was reported previously [64], showing that autophagy and apoptosis are closely connected [64,65,66]. Autophagy may either inhibit or lead to apoptosis. In our experimental system, inhibition of autophagy by chloroquine led to increased levels of the apoptotic marker, cleaved caspase-3, in control (V) vs. heparanase overexpressing (H) cells. This suggests that heparanase plays a protective function in podocytes exposed to combined treatment with Adriamycin and chloroquine. Thus, unlike its deleterious properties in disease states such as cancer and inflammation, heparanase seems to play a favorable role in podocytes, protecting the cells from harsh environmental conditions.

In conclusion, the question of “whether heparanase contributes to the pathogenesis of glomerular diseases” is still not fully answered. A thorough investigation by Garsen et al. suggested that endothelin 1 induces heparanase expression and release from podocytes, resulting in damage to the endothelial glycocalyx and proteinuria in acute glomerulonephritis experimental models [24]. However, our findings propose that stable overexpression of heparanase in podocytes may exert cytoprotection via enhancement of podocyte viability, lysosomal activity and autophagic flux, pari passu with attenuation of apoptosis, elicited early after the induction of injury in experimental model of Adriamycin-induced nephropathy. Therefore, stable expression of heparanase in podocytes may lead to different biological behaviors. Consistent with our observations, two recent studies reported that heparanase protected *hpa*-TG mice from streptozotocin-induced diabetes and attenuated heart injury from ischemia/reperfusion or toxic insults [67,68].

Appropriate concerns might be raised because heparanase contributes to the pathogenesis of cancer and certain inflammatory diseases [9,69]. Assuming that podocyte protection by heparanase involves non-canonical functions of this protein, effects may be tissue-specific and may not necessarily entail heparanase systemic activation. Further deciphering the interplay between heparanase expression and resistance of glomerular cells to injury and insights gleaned from additional unbiased studies, would relieve concerns about unwanted effects of heparanase and may uncover additional downstream or interacting cellular and molecular pathways that would be translated into novel therapies, regardless of the presence or absence of heparanase in kidney disease.

## 4. Materials and Methods

### 4.1. Cell Lines and Heparanase Overexpression

Undifferentiated, conditionally immortalized human podocyte cells (AB8/13 cell line, a kind gift of Prof. Moin Saleem, Bristol, UK) were cultured in RPMI 1640 medium (Sigma, St. Louis, MO, USA) supplemented with 10% fetal bovine serum (Sigma), 1% Insulin–Transferrin–Selenium (ITS, Invitrogen, Amsterdam, The Netherlands), and 1% penicillin/streptomycin (Biological Industries, Beit HaEmek, Israel) at 33 °C in 5% CO_2_ incubator. To induce differentiation, cells were transferred to 37 °C for 14 days, as previously described [31].

To overexpress the human heparanase within cells, undifferentiated AB8/13 podocyte cells, maintained at 33 °C, were infected with pLenti6/V5-DEST control empty vector (V) or heparanase gene construct (H), selected with blasticidin (10 μg/mL; Invitrogen), and expanded. Experiments were performed using cells from passages 25–34.

### 4.2. Adriamycin-Induced Injury and Cell Survival

Survival of cells was determined by the methylene blue colorimetric assay [70]. For calibration, AB8/13 podocytes overexpressing heparanase, Hepa (H) cells, or their mock-infected control (V) cells, were seeded in quadruplicates of 600–20,000 cells per well in 96-well plates. After 12 h, adherent cells were fixed with 4% formaldehyde. For Adriamycin treatment, 7500 cells were plated per well, allowed to differentiate for 14 days, then treated with Adriamycin (Teva, Tel Aviv, Israel) for 24 h and fixed. Methylene blue (Sigma) staining, absorption, and conversion to cell number were performed as previously described [70]. Results were expressed as percent of control for each cell type (% of control = treated cell number/untreated cell number). Of note, other measures of cell number were found inappropriate because of overlap between the fluorescence spectra of the indicator and Adriamycin autofluorescence.

### 4.3. Cell Viability Assay

Hepa (H) cells and their control (V) cells were seeded in quadruplicates of 20,000 cells per well in 96-well plates, allowed to differentiate for 14 days, then treated with Adriamycin 0, 0.0625, 0.125, 0.25, 0.5, 1, 2, and 4 μg/mL for 24 h. Cell viability was determined using the Neutral Red Assay Kit-Cell Viability/Cytotoxicity (Abcam, Cambridge, UK) according to the manufacturer’s instructions.

### 4.4. Experimental Animals

Male homozygous *hpa*-TG mice, in which the human heparanase gene is driven by a constitutive β-actin promoter in a BALB/c genetic background, were applied [38]. Male wild-type (*wt*) BALB/c mice, 10–12 weeks old, were purchased from Harlan Laboratories (Jerusalem, Israel) and served as controls. Mice were maintained under conventional pathogen-free conditions, in a temperature-controlled room, and fed with standard mouse chow and tap water ad libitum. The study protocol was approved by the Technion Animal Inspection Committee (IL-0240217). All experiments were performed in accordance with the institutional guidelines and regulations. *hpa*-TG or *wt* mice were randomly assigned to the following experimental groups, 5 mice each: C—animals injected with vehicle (PBS) serving as control; A—experimental Adriamycin-induced nephropathy (10 mg/kg, i.v.), sacrificed after 3 days; Q—mice injected with chloroquine (40 mg/kg, i.p., Sigma), 7 h before being sacrificed; and AQ—mice injected with both Adriamycin and chloroquine.

### 4.5. mRNA Expression Analysis

Gene expression was analyzed by quantitative reverse transcription PCR (RT-qPCR), using the Rotor-Gene 6000 instrument (Corbett Life Science, Mortlake, Australia). Total RNA was isolated from podocytes using EZ-10 DNAaway RNA Miniprep kit (Bio Basic Canada Inc., Markham, ON, Canada), according to manufacturer instructions. Real-time PCR was performed following cDNA synthesis (qPCRBIO cDNA Synthesis Kit, PCR Biosystems) using qPCRBIO SyGreen Blue Mix (PCR Biosystems), according to the manufacturer’s instructions. The following PCR primers were used: ***hB2M*** fw 5′ TGC TGT CTC CAT GTT TGA TGT ATC T 3′, rev 5′ TCT CTG CTC CCC ACC TCT AAG T 3′; ***hATG5*** fw 5′ GCA AGC CAG ACA GGA AAA AG 3′, rev 5′ GAC CTT CAG TGG TCC GGT AA 3′; ***hATG12*** fw 5′ CGA ACA CGA ACC ATC CAA GG 3′, rev 5′ TCA CTG CCA AAA CAC TCA TAGA 3′; and ***BECN1*** fw 5′ ATG CAG GTG AGC TTC GTG TG 3′, rev 5′ CTG GGC TGT GGT AAG TAA TGG A 3′. Gene expression was calculated using a standard curve which relates Ct to concentration. Gene expression of the target sequence was normalized to a housekeeping gene, *B2M*. Results of Hepa (H) cells were expressed relative to control mock-infected (V) cells, which was arbitrarily assigned a value of 1.

### 4.6. Western Blotting

Cells, seeded on 6-well plates or 100 mm plates, were lysed in RIPA buffer (EMD Millipore Corp, Billerica, MA, USA), containing a protease inhibitor cocktail (Sigma, 1:50 dilution) and phosphatase inhibitor cocktail 3 (Sigma, 1:100 dilution) at 4 °C. Equal amounts of extracted proteins (25 μg) were resolved by electrophoresis on 10–17% SDS-polyacrylamide gel and were transferred to nitrocellulose or PVDF membranes using either BioRad Trans-Blot^®^ Turbo™ system or wet transfer. The membranes were incubated in blocking buffer, TBS-T (Tris-buffered saline and 0.1% Tween 20) containing 5% (*w*/*v*) BSA for 1 h, followed by overnight incubation at 4 °C, with the appropriate primary antibody: polyclonal rabbit anti-heparanase (INS-26-2-0000-11, InSight Biopharmaceuticals Ltd, Rehovot, Israel, 1:1000), anti-cleaved caspase-3 (9661, Cell Signaling, Danvers, MA, USA, 1:1000), anti-LC3 (L8918, Sigma Aldrich, 1:1000), anti-phospho Beclin (Thr119) (ABC118, MilliporeSigma™, 5 μg/mL), anti-PARP (9542, Cell signaling, 1:500), anti-SQSTM1/p62 (5114, Cell signaling, 1:1000), anti-GRP78 (ab109659, abcam, Cambridge, UK, 1:1000), anti-phospho p70 S6 Kinase (Thr389) (9205, Cell signaling, 1:500), or mouse monoclonal anti-ATG12 (sc-271688, Santa Cruz, Dallas, TX, USA, 1:500) and anti-GAPDH (sc-47724, Santa Cruz 1:000) antibodies. After washing with TBST, the membranes were reacted for 1–2 h at room temperature (RT) with a secondary horseradish peroxidase-conjugated IgG (Goat anti-rabbit, 7074; Horse anti-mouse, 7076, Cell Signaling), diluted 1:3000. The immunoreactive proteins were visualized with an enhanced chemiluminescence substrate (WesternBright, Advansta, San Jose, CA, USA) and analyzed using luminescent image analyzer LAS-4000 (Fujifilm, Tokyo, Japan). Densitometry analysis was performed using the ImageQuant software (GE Healthcare Bio-Sciences, Piscataway, NJ, USA). Protein semi-quantitation for each experimental repetition was calculated from the western blot raw data, normalized to GADPH for each well, and averaged across replicas.

### 4.7. Immunofluorescence

#### 4.7.1. In Vivo

Whole kidneys were harvested, quickly frozen in liquid nitrogen, and 4 μm thick cryostat sections were placed on silane-coated glass slides and dried at room temperature. Sections were first mildly permeabilized with 0.05% saponin in PBS, 10 min at RT, washed with PBS, and then fixed with cold methanol for 10 min. Slides were washed and blocked with 10% normal serum in PBS at RT for 1 h and incubated with the primary antibodies: guinea pig anti-nephrin (BP5030, ORIGENE, Rockville, MD, USA, 1:100) and rabbit anti-LC3 (L8918, Sigma Aldrich, 1:200), at RT for 2 h. Then, slides were washed and incubated with secondary antibodies: AlexaFluor^®^ 488 goat anti-rabbit IgG (1:200, IR 111-545-144, Jackson ImmunoResearch Laboratories, West Grove, PA, USA) and DyLight^®^ 650 guinea pig anti-goat (1:200, A60-110D5, Bethyl Laboratories, Montgomery, TX, USA) at RT for 1 h. Nuclei were counterstained with 4′, 6-diamidino-2-phenylindole (DAPI) Fluoromount–G (SouthernBiotech, Birmingham, AL, USA). Images were acquired using laser scanning confocal inverted microscope (Zeiss LSM700, Oberkochen, Germany, X63 oil immersion objective). The 3D digital images with Z-stacks comprising up to 40 images were captured with a highly sensitive Axiocam ICc3 camera, controlled by Axiovision software version 4.8. Confocal pinhole was set to 1 Airy unit. Image size was 512 × 512 pixels.

Quantitative image analysis of number, size, and fluorescence intensities of LC3-positive puncta, a gold-standard assay for assessing autophagosomes in cells, was performed using pre-determined size and intensity thresholds functions in Imaris software (version 9.8.2, Oxford Instruments, Abingdon, UK).

#### 4.7.2. In Vitro

Podocytes cells were plated on coverglass and incubated at 37 °C for 14 days. Cells were then treated with ADR to a final concentration of 0.5 μg/mL and 50 μM chloroquine (Sigma, C6628) for 2 hrs. Treated cells were permeabilized and fixed with 4% Paraformaldehyde (PFA) containing 5% sucrose and 0.1% Triton X-100 for 5 min at RT and re-fixated with 4% PFA containing 5% sucrose for 25 min. After washing with PBS, coverslips were blocked with IF buffer (130 mM NaCl, 7 mM Na_2_HPO_4_, 3.5 mM NaH_2_PO_4_, 7.7 mM NaN_3_, 0.1% BSA, 0.2% Triton X-100, 0.05% Tween20) supplemented with 10% donkey serum for 1 h and incubated overnight at 4 °C with either antibody. The primary antibodies used were as follows: rabbit monoclonal antibody to LAMP1-conjugated with Cy3 (1:100, Abcam, ab67283) and rabbit antibody to LC3 (1:15-0, Sigma-Aldrich, L8918). The cells were washed three times with PBS for 15 min each and incubated for 60 min with donkey anti-rabbit conjugated to Alexa Fluor 647 (ab150067, Abcam), washed as above, and nuclei were counterstained with 4′, 6-diamidino-2-phenylindole (DAPI) Fluoromount–G (SouthernBiotech, Birmingham, AL, USA). Images were acquired using the Zeiss LSM 880 laser scanning confocal attached to Axio Examiner Z1 upright microscope (Zeiss Germany), with X63 NA1.4 oil immersion objective and lasers line 405 nm, 488 nm, 561 nm, and 633 nm. The LSM 880 was controlled by ZEN Black 2.3 (Zeiss Germany).

Quantitative image analysis of LC3 colocalization with LAMP1 staining was performed by using Imaris software version 9.8.2 (Oxford Instruments). Each confocal image was split into single-color channels and segmented to select the contribution of the green and red fluorescence, respectively. Background signal was excluded by setting a threshold in both color channels, and the analysis of colocalization was manually performed. For each experiment, at least 10 cells were scored for each condition. 

### 4.8. Statistical Analysis

All experiments were repeated at least three times. Data were expressed as means of repeated measurements ± standard error of the mean (SEM). Differences between groups were analyzed using a two-tailed Student’s *t*-test. *p*-values of 0.05 or less were considered statistically significant.

## Data Availability

All relevant data are included in the manuscript, Supplementary Materials, or will be made available upon request to the corresponding author.

## Supplementary Materials

The following are available online at https://www.mdpi.com/article/10.3390/ijms232012691/s1.
