# Supplementary material for: Heparanase Increases Podocyte Survival and Autophagic Flux after Adriamycin-Induced Injury
Source: Int J Mol Sci. 2022 Oct 21;23(20):12691. doi: 10.3390/ijms232012691 (PMC9604275; doi:10.3390/ijms232012691)

Supplementary Material to

Heparanase Increases Podocyte Survival and Autophagic Flux after Adriamycin-Induced Injury

Hanan Abu-Tayeh Suleiman [1,†], Shereen Said [2,†], Haya Ali Saleh [2], Aviva Gamliel-Lazarovich [1], Eyas Haddad [2], Irina Minkov [3], Yaniv Zohar [2,3], Neta Ilan [4], Israel Vlodavsky [4], Zaid Abassi [2,5], and Suheir Assady [1,2,*]

[1] Department of Nephrology and Hypertension, Rambam Health Care Campus, Haifa 3109601, Israel.
[2] Rappaport Faculty of Medicine, Technion - Israel Institute of Technology, Haifa 3109601, Israel.
[3] Department of Pathology, Rambam Health Care Campus, Haifa 3109601, Israel.
[4] Cancer and Vascular Biology Research Center, Rappaport Faculty of Medicine, Technion, Haifa 3109601, Israel.
[5] Department of Laboratory Medicine, Rambam Health Care Campus, Haifa 3109601, Israel.
* Correspondence: s_assady@rambam.health.gov.il; Tel.: +972-4-777-2852.
† These authors contributed equally to this work.

Supplementary Figure S1. Overexpression of heparanase in human podocytes. Undifferentiated AB8/13 podocytes (P) were infected with empty pLenti6/V5-DEST virus (V) or human heparanase cDNA (H). Differentiated cells were exposed to 0.5 and 1 μg/ml Adriamycin (ADR) for 24 hours. The expression of heparanase was detected by immunoblotting (10% SDS-PAGE, 25 μg protein), using polyclonal rabbit anti-heparanase (INS-26-2-0000-11, InSight Biopharmaceuticals; 1:1000). Monoclonal anti-beta actin (ab197277, Abcam; 1:4000) was used to detect the housekeeping protein.

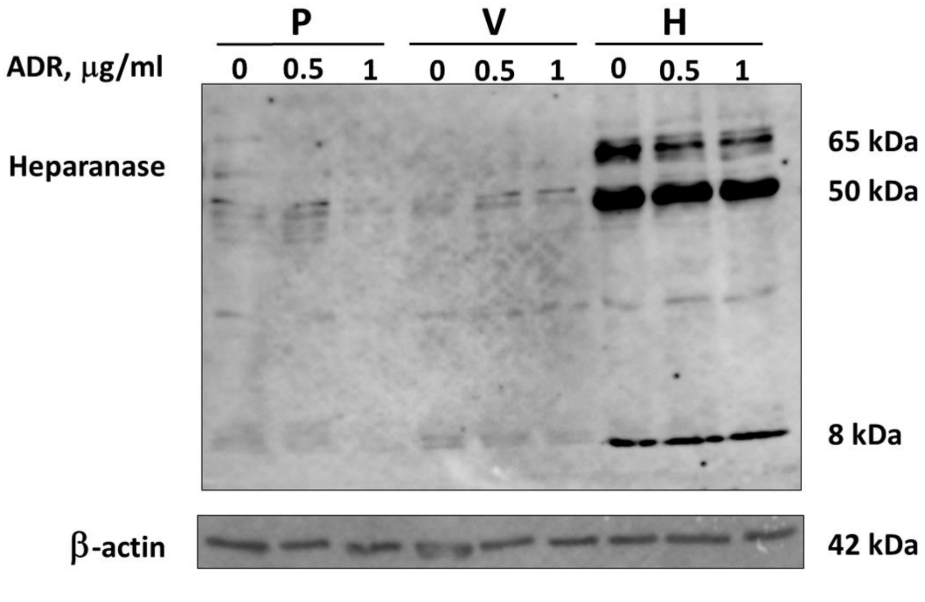

Supplementary Figure S2. Podocyte cell survival after H_2O_2-induced oxidative stress is associated with increased autophagic flux in heparanase overexpressing cells.

(a) Podocytes, V and H cells, were seeded in a 96-well plate. Differentiated podocytes were exposed to different concentrations of H_2O_2 for 24 hours. Cell number was determined using methylene blue assay. Results were expressed as percent of control for each cell type, i.e., the ratio of treated cell number to control untreated cells. The results are expressed as the means ± SEM of 3 independent experiments, quadruplicates in each experiment. Insert in (a): Cell survival at 0.5 mM H_2O_2. (b) V and H podocytes were treated with 0.5 mM H_2O_2, 50 μM Chloroquine (Q), or a combination of 50 μM chloroquine ± 0.5 mM H_2O_2 for 14 hours. Whole-cell lysates were subjected to SDS-PAGE and immunoblotting for LC3 with GAPDH as a loading control. V, Mock-infected podocytes; H, heparanase overexpressing podocytes.

* $p \leq 0.05$.

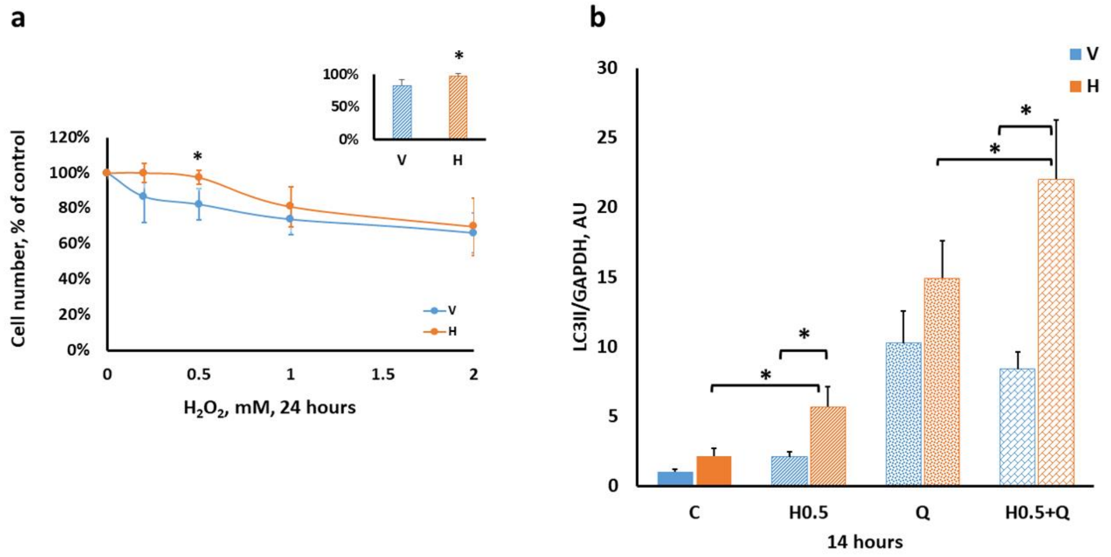

Supplementary Figure S3. A time-dependent increase in LC3II after Adriamycin and chloroquine treatment is more prominent in heparanase overexpressing cells. Mock-infected (V) and heparanase overexpressing (H) differentiated podocytes were grown in RPMI (C) or treated with 0.5 μg/ml Adriamycin (ADR) ± 50 μM chloroquine (Q) for the indicated time intervals. Whole-cell lysates were subjected to SDS-PAGE and immunoblotting for LC3 with GAPDH as a loading control. Representative immunoblots are shown; *n* = 3 independent experiments.

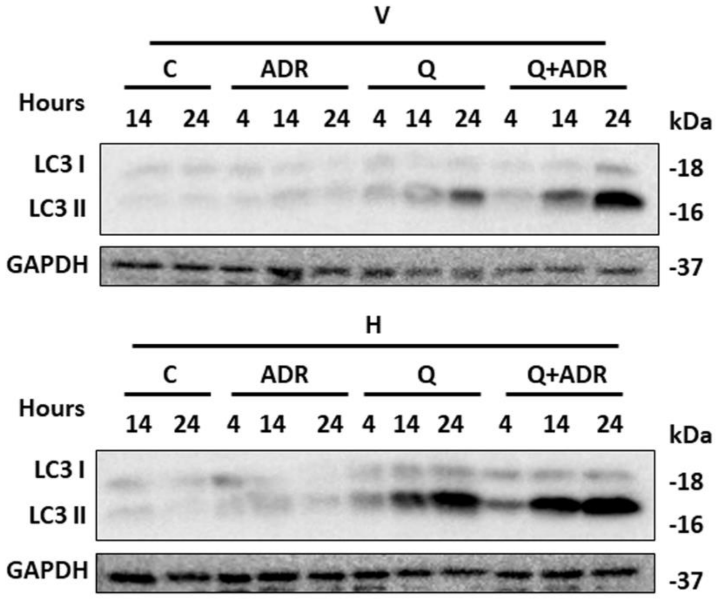

Supplementary Figure S4. Roneparstat decreases Atg12 levels in heparanase overexpressing podocytes. Differentiated heparanase overexpressing podocytes were treated with 0.5 μg/ml Adriamycin (ADR) ± 100 μg/ml Roneparstat (SST0001, heparanase inhibitor) for 2 hours. Whole-cell lysates were subjected to SDS-PAGE and immunoblotting for Atg12 with GAPDH as a loading control. (a) Representative immunoblots of Atg12. (b) Quantification of Atg12 (21KDa) normalized to GAPDH. Results are expressed as the means ± SEM, *n* = 4 independent experiments. * $p \leq 0.05$, ** $p \leq 0.01$, *** $p \leq 0.001$.

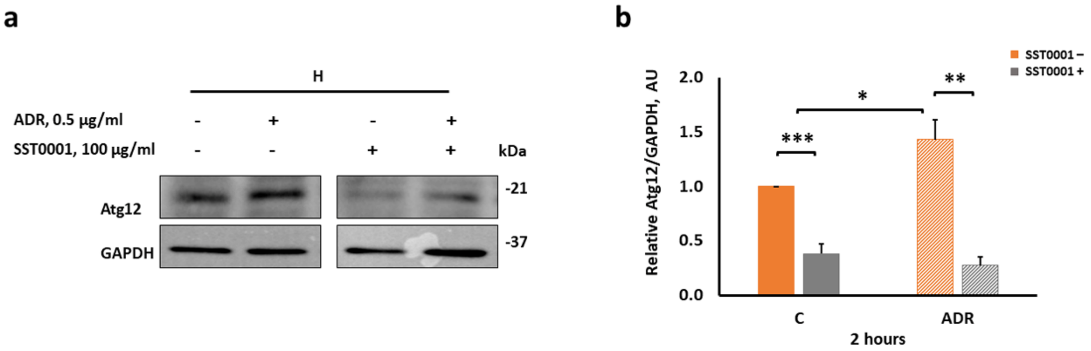

Supplementary Figure S5. Adriamycin induces apoptosis in cultured human podocytes. Differentiated AB8/13 podocytes were treated with Adriamycin at the indicated concentrations (a) for 24 hours, or with 0.5 μg/ml Adriamycin for the indicated time (b). Whole-cell lysates were subjected to SDS-PAGE and immunoblotting for cleaved caspase-3 with GAPDH as a loading control. Representative immunoblots are shown.

a

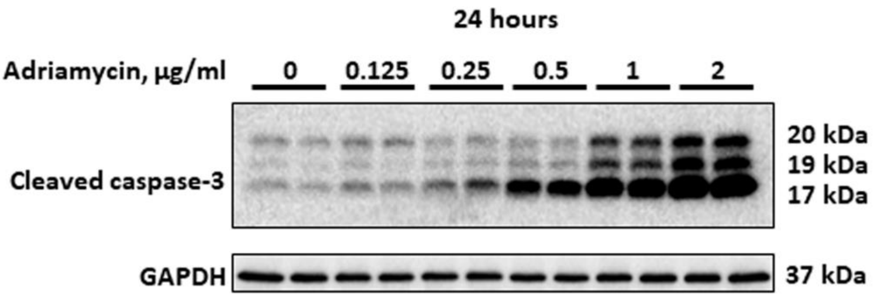

b

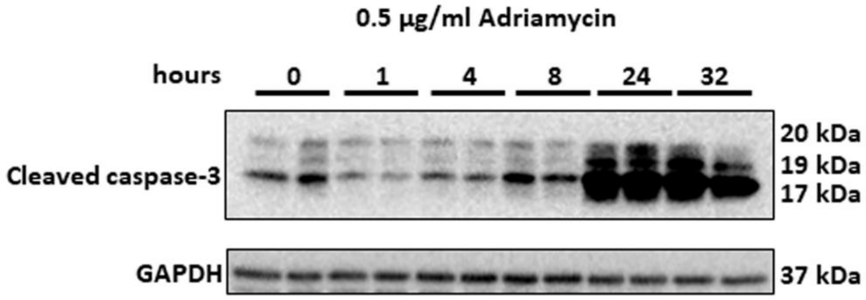

Supplement: Supplementary file 1 [file ijms-23-12691-s001.zip › ijms-1949646-supplementary.pdf]
